# Oblique, forward, and lateral directions reach test distances in young adults, and concurrent validity of these tests with the center of pressure excursion in assessing the limits of stability

**DOI:** 10.1016/j.heliyon.2024.e24591

**Published:** 2024-01-17

**Authors:** Jaya Shanker Tedla, Devika Rani Sangadala, Ravi Shankar Reddy, Kumar Gular, Venkata Nagaraj Kakaraparthi, Snehil Dixit, Ahlam Mohammed Alamri, Akshatha Nayak, Gopal Nambi, Bhaskar Reddy Ponneru

**Affiliations:** a Department of Medical Rehabilitation Sciences, College of Applied Medical Sciences, King Khalid University, Alfara, Abha, Kingdom of Saudi Arabia; b Department of Rehabilitation Health Services, Armed Forces Hospital Southern Region, Khamis Mushayat, Kingdom of Saudi Arabia; c Department of Physiotherapy, Kasturba Medical College, Mangalore, Manipal Academy of Higher Education, Manipal, Karnataka, India; d Department of Health and Rehabilitation Sciences, College of Applied Medical Sciences, Prince Sattam Bin Abdulaziz University, Al-Kharj, Kingdom of Saudi Arabia; e Department of Physical Therapy, King Khalid Hospital, Najaran, Kingdom of Saudi Arabia

**Keywords:** Balance, Center of pressure, Limits of stability, Functional activities, Reach test, Concurrent validity

## Abstract

Limits of stability is required to perform functional activities and other advanced tasks of life without losing balance, and assessment of limits of stability is essential in clinical practice. Forward, Lateral, and Oblique direction reach tests are clinical balance tests that assess limits of stability, and these reach distances in various directions may be symmetrical or asymmetrical. The aim was to establish the symmetry between various reach distances on three reach tests and to establish the concurrent validity of oblique, forward, and lateral direction reach test distances with limits of stability measured by the Iso Free machine of TecnoBody company. Methods: The measurements of oblique, forward, and lateral reach tests and limits of stability excursions of center of pressure were taken in eight directions on Iso Free machine of Techno Body in fifty typical college-going young adults who were recruited through convenience sampling. Pearson correlation test was used to find the relationship between forward, lateral, and oblique direction reaches and limits of stability in forward, lateral, and oblique directions. Regression analysis was used to find the factors influencing the forward, lateral, and oblique reach tests. Results: The reach distances were symmetrical, and out of the three tests, the lateral and oblique direction reach tests have a moderate positive correlation with limits of stability test in lateral and oblique directions with an r-value of 0.569 (p < 0.001) and 0.50 (p < 0.001) respectively. A Significant standardized beta value of 0.49 (p < 0.05) for the oblique direction reach test with total stability limits. Conclusions: The oblique and lateral direction reach tests are correlated with their respective center of pressure excursion. However, the oblique direction reach test moderately correlated with the total limits of stability scores. Forward reach distances were more in number followed by oblique and lateral reach distances among young Saudi adults.

## 1 Introduction

### 1.1 Background of the study

Balance is the ability to align several body segments and produce multi-joint movements by effectively controlling the Center of Pressure (COP) within and outside of the base of support [1,2]. It is highly task-specific and controlled by the appropriate function of multiple complex body systems, such as the sensory, vestibular, and neuromuscular systems, as well as biomechanical and neuropsychological factors [3]. Because balance is task-oriented, it is classified as static (maintaining a stable position, such as sitting or standing), dynamic (walking), proactive (anticipating a predicted disturbance, such as crossing or walking), and reactive (anticipating a predicted disturbance, such as crossing) [4,5].

Balance is essential in performing different functional activities [6]. Some functional activities, such as reaching for objects while preparing a meal or reaching for a door handle, require the limits of stability (LOS) [6,7]. Maintaining the core stability and the extent to which the body can move outside the base of support without taking a step and changing the base of support is known as the LOS [3]. In other terms, functional LOS can be defined as the percentage of the base of support in which individuals can extend their COP [8].

In healthy individuals, motor performance, accuracy, speed, and balance control can be affected by sleep disturbance. Sleep deprivation is a state caused by inadequate sleep quantity or quality. Two types of sleep deprivation are acute sleep deprivation and chronic sleep deprivation. Chronic sleep deprivation is the most common disorder that occurs in the general population. These different types of sleep disorders may have an impact on balance control [9]. Balance is affected by various pathologies involving the nervous, sensory, and musculoskeletal systems. Individuals with balance deficits have difficulty performing functional reaching activities [8,10].

Therefore, it is crucial to assess balance in the context of the LOS for rehabilitation professionals [11]. There are different methods for measuring the functional LOS in clinical practice. The gold standard approach is the use of a force plate to measure COP displacement. LOS measurement using a force plate on Iso-Free (TecnoBody) is one of the recent methods utilized [[12], [13], [14]]. Simple, valid, and reliable tests are also available for measuring the functional LOS in clinical settings. The forward reach test (FRT) is a valid and reliable test developed by Duncan et al. that measures the LOS in the forward direction [15,16]. The lateral reach test (LRT) developed by Brauer et al. measures the LOS in the mediolateral direction [17]. Meanwhile, Newton developed the multi-directional reach test, which is valid and reliable in assessing the LOS in the forward, backward, and right and left lateral directions [18].

The functional LOS requires stability not only in the forward, backward, and lateral directions but also in the oblique direction, as humans perform most activities of daily living in the oblique direction, including kitchen activities and office desk tasks. Based on this concept, Tedla et al. developed the oblique direction reach test (ODRT), which measures the LOS in the oblique direction. Their study proved the test–retest reliability and concurrent validity of this test with the FRT and LRT [6]. Moreover, the symmetry of the reach distances among these three directions reach tests was not commented on before and would like to see it in the current study.

### 1.2 Statement of problem

• The gold standard method of measuring limits of stability is COP excursions using a force platform. The concurrent validity of the ODRT with the COP excursions has yet to be established.
• There is a shortage in the literature regarding which among the FRT, LRT, and ODRT will be greater and show excellent correlation in comparison with COP excursion.

### 1.3 Aims and objectives of the study

• The principal aim of the current study was to evaluate the concurrent validity of the ODRT with the measurement of the COP excursion using the Iso-Free machine of Techno Body company.
• The secondary aim was to determine which reach test distance correlates well with the COP excursion.

### 1.4 Practical importance

• Suppose this study proves the validity of the ODRT with the COP excursions. In that case, ODRT is a simple and feasible test to measure the limits of stability in an oblique direction in the clinical setting.

## 2 Materials and methods

### 2.1 Sampling and determination of sample size

This cross-sectional study was approved by the research ethics committee at King Khalid University (REC # 2016-08-29). In this cross-sectional study, we recruited 50 healthy college-aged young adults (men: 32; women: 18) (mean age: 22.18 years) using convenient sampling. The sample size for this research was estimated utilizing the online sample size calculator at https://clincalc.com/Stats/SampleSize.aspx website. We used the one study group versus population option, and since the reach test values are presented in centimeters, we selected the software's continuous variable option. The known population's expected means and standard deviations (SDs) were obtained from previously published literature [6]. The anticipated oblique reach distance was kept at 25 cm. The power was 80 %, and the alpha value was 0.05. We obtained a sample size of 46 but decided on a sample size of 50, considering a dropout rate of 10 %.

### 2.2 Procedure

The sample was approached after obtaining ethical approval from the institutional research ethics committee of King Khalid University (approval number REC 2021-08-29). The sample was selected from students at the College of Applied Medical Sciences. Announcements were made in the classrooms and on the notice boards of the college regarding the study details, and interested candidates were then approached. The study details were explained to the participants, and their written informed consent was obtained. The demographic characteristics, such as sex, age, height, mass, and communication details, were recorded. Height was measured in meters using a stadiometer and mass in kilograms using a weighing machine. The body mass index was calculated as mass in kilograms divided by height in meters squared using the statistical software.

**Fig. 1:**
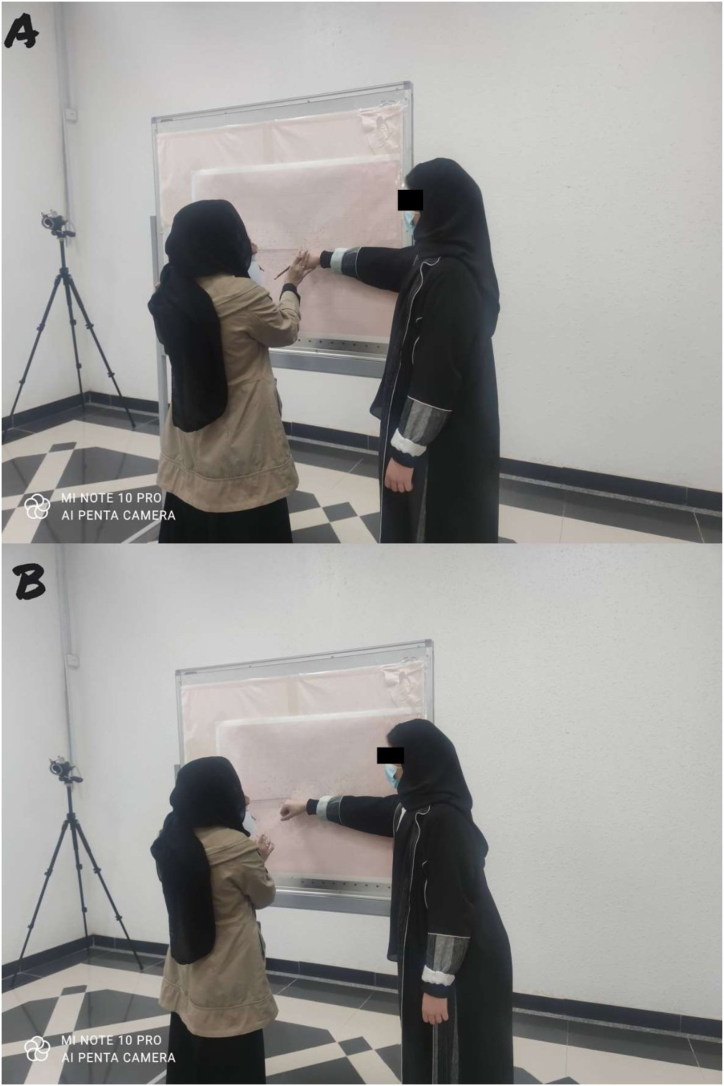
Demonstrating procedure of forward reach beginning (A) and ending (B) by a participant and measurement collection by a therapist.

**Fig. 2:**
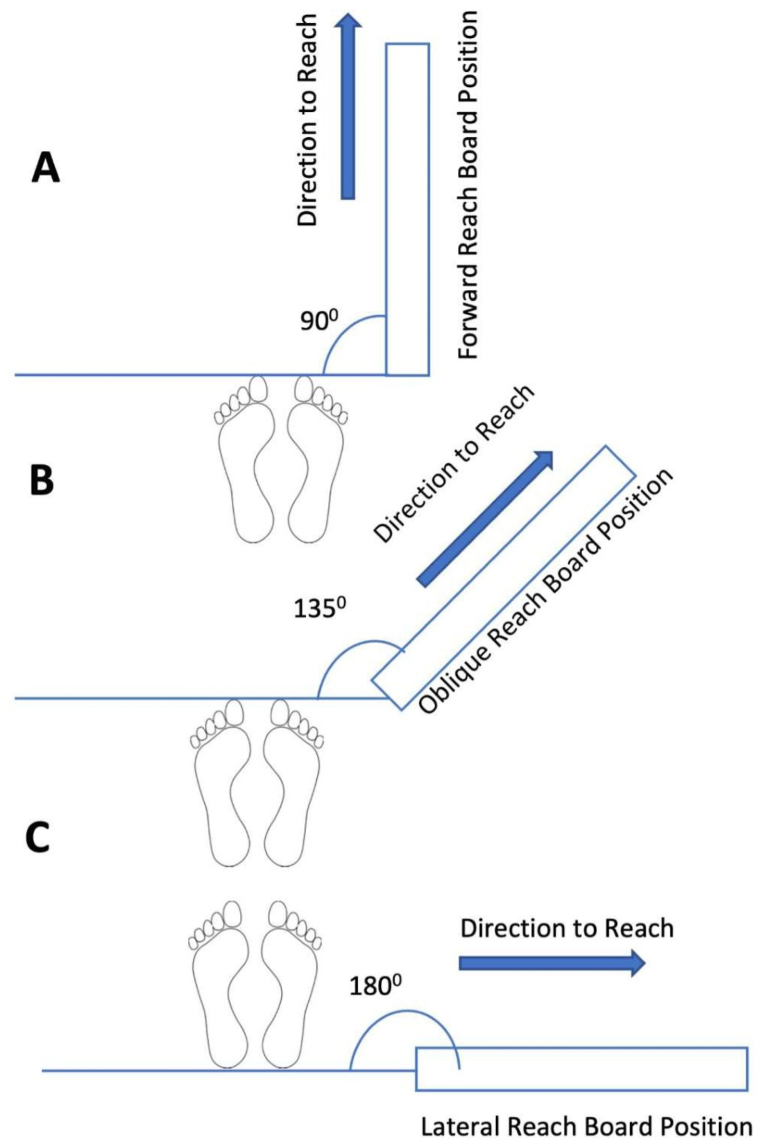
Depicting the foot posture and board position in forward (A), oblique (B), and lateral (C) direction reach tests.

The FRT, LRT, and ODRT were conducted in an assessment room. A graph paper was fixed on a movable whiteboard, as shown in Fig. 1(A and B), and the participants were positioned in such a way that their feet were separated by shoulder width and the tip of the foot touched a horizontal line, as demonstrated in Fig. 2(A-C). In the FRT, the participants leaned in the forward direction, as shown in Fig. 1(A and B). In the ODRT and LRT, the participants leaned in the oblique and lateral directions, as demonstrated in Fig. 2 (B and C). The upper-limb position was 90° flexion for forward reach, 90° flexion and abduction for oblique reach, and 90° abduction for lateral reach. The tip of the third metacarpal of a closed fist was used as the corresponding point for marking on the graph chart. The distance between the starting and ending points on the graph chart was considered for the reach distance measurement. The procedures have been detailed in previously published literature [6,19,20].

**Fig. 3:**
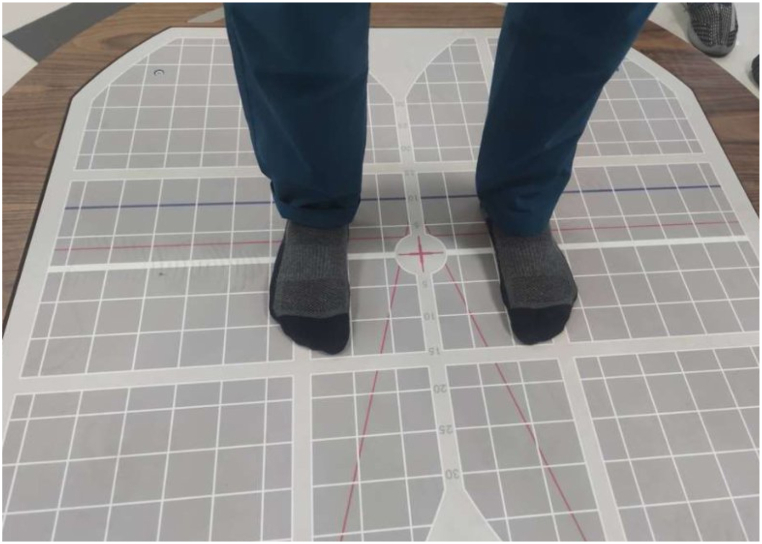
The starting foot position on the force platform of the Iso Free machine (TecnoBody).

**Fig. 4:**
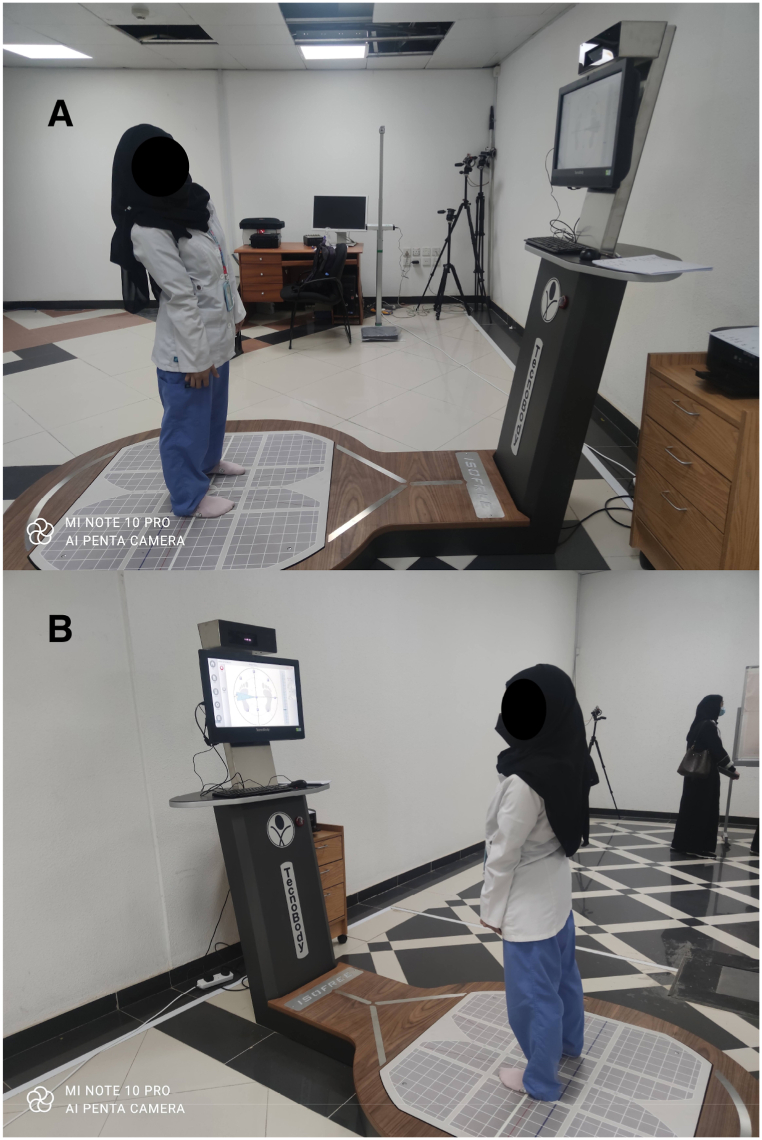
Showing the limits of stability assessment on the Iso-free machine of TecnoBody company.

**Fig. 5:**
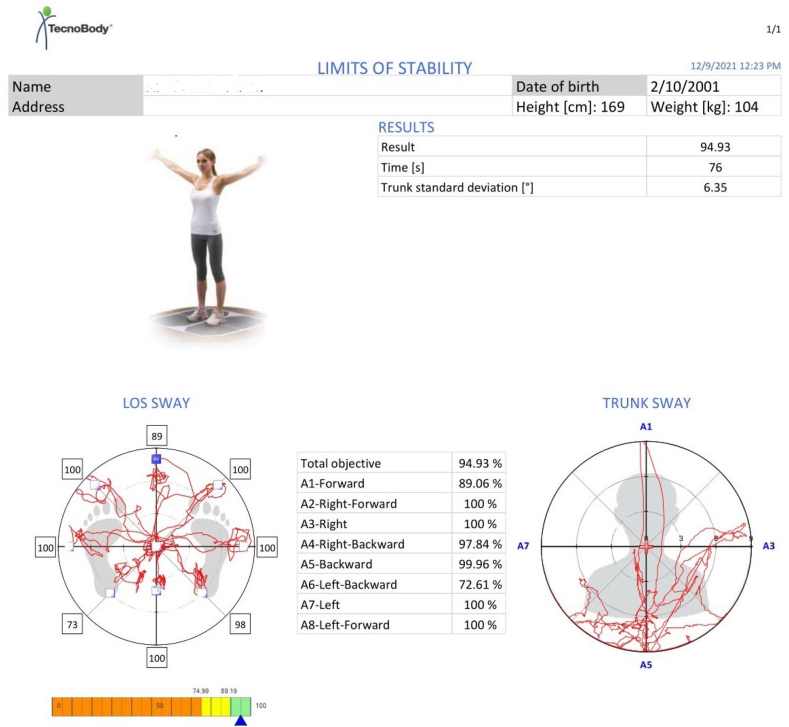
Results of the limits of stability assessment, including the center of gravity sway graph, trunk sway graph, eight-direction balance percentages, total balance percentage, test duration, and demographic data.

The LOS test was conducted on the Iso-Free machine of Techno Body company. After the required data were entered into the software, the participants were positioned on the force platform in the prescribed format of the machine; the details are shown in Fig. 3. The LOS was measured in eight directions: forward (A1), right forward (A2), right (A3), right backward (A4), backward (A5), left backward (A6), left (A7), and left forward (A8). The therapist demonstrated in one direction in front of all participants, and the actual test was then performed. The machine screen provided visual feedback to the participants, and they could monitor their COP as a black dot on the white screen with the desired direction shown as a yellow box. The participants moved their body toward the desired direction and ensured the black dot coincided with the yellow box. Once they completed one direction, the machine prompted another direction randomly. The testing position details are displayed in Fig. 4(A and B). When all eight directions were completed, the test was marked as complete, and the results were displayed on the screen and saved in a PDF file. The COP excursion in various directions is further detailed in Fig. 5. The LOS in multiple directions and the total LOS were described in percentages. The total possible maximum percentage was 100. Values between 90 and 100 indicated sports-level performance with excellent balance; between 75 and 90, normal balance; and below 75, abnormal balance. After the measurements, we compared the reach distance with the LOS to obtain the concurrent validity of the reach tests.

### 2.3 Statistical analysis

The software used for the analysis was SPSS version 24. Univariate analysis of all the mean, standard deviation, minimum, maximum, and range variables was done using descriptive statistics. The correlation between reach distances and limits of stability was done using Pearson correlation. Regression analysis used linear regression to identify the factors affecting reach distances**.**

## 3 Results

**Fig. 6:**
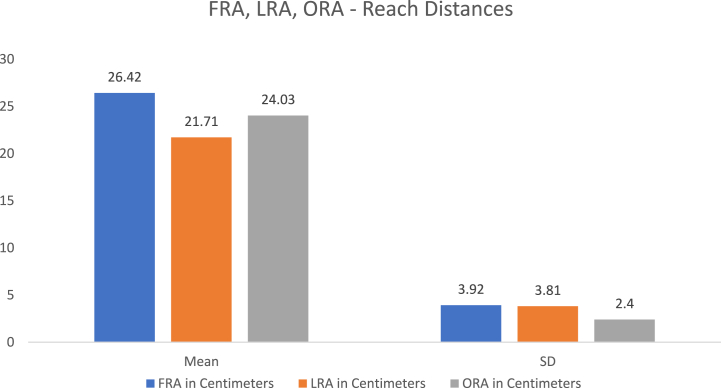
Graph showing the Averages of Forward, lateral, and oblique Reach distances.

The mean age of the participants was 22.18 years, with an SD of 0.66 years. The mean ± SD of the demographic characteristics, such as height, mass, and body mass index, of the entire sample is presented in Table 1. The mean ± SD of the forward reach LOS was 87.88 ± 11.70; right forward oblique reach LOS, 99.31 ± 2.28; and right lateral reach LOS, 97.96 ± 5.99. The total LOS and trunk SD during the LOS test are presented in Table 2. The mean ± SD of the forward, right lateral, and right oblique reach distances, which were measured using the graph paper method, are also shown in Table 2. & Fig. 6, where the forward reach distance was more than the oblique and lateral reach directions.

**Table 1:** Details of demographic characteristics in terms of mean, standard deviation, minimum, and maximum.

Variables	Mean	Standard Deviation	Minimum	Maximum
Age	22.18	0.66	21.00	23.00
Height in meters	1.66	0.07	1.48	1.80
Mass in KG	71.14	18.33	44.00	119.00
BMI in KG/Mt^2^	25.90	6.76	18.20	45.34

**Table 2:** Limits stability parameters and reach mean, standard deviation, minimum, and maximum distances.

Variables	Mean	Standard Deviation	Minimum	Maximum
Total Limits of Stability Score %	95.60	3.13	87.66	100.00
A1: Forward reach LOS %	87.88	11.70	62.95	100.00
A2: Right forward ODRT LOS %	99.31	2.28	89.98	100.00
A3: Right Lateral reach LOS %	97.96	5.99	74.56	100.00
Total time taken for LOS testing in sec	78.01	3.55	72.00	86.00
Trunk Standard Deviation in degrees	7.70	3.46	2.71	15.99
FRT1 in Centimeters	25.63	4.06	17.50	33.00
FRT2 in Centimeters	26.74	4.24	19.00	36.00
FRT3 in Centimeters	26.90	3.86	21.00	35.00
FRA in Centimeters	26.42	3.92	20.67	34.33
LRT1 in Centimeters	21.41	3.85	12.00	30.00
LRT2 in Centimeters	21.53	3.90	11.00	33.00
LRT3 in Centimeters	22.20	4.06	12.00	33.00
LRA in Centimeters	21.71	3.81	12.33	32.00
ORT1 in Centimeters	23.63	2.42	19.00	30.00
ORT2 in Centimeters	23.93	2.90	18.00	31.00
ORT3 in Centimeters	24.52	2.89	19.00	29.00
ORA in Centimeters	24.03	2.40	18.67	29.00

**Note**: KG: Kilogram, Mt: Meter, LOS: Limits of stability, FRT: forward reach test, LRT: Lateral reach test, ORT: Oblique direction reach test, FRA: functional reach test average, LRA: Lateral reach average; ORA: Oblique direction reach test average.

Table 3 shows the relationship between the forward, lateral, and oblique reach distances and LOS. There was a non-significant correlation of the forward reach distance with the age, height, mass, and BMI, as well as the forward, right lateral, and right forward oblique reach LOS and the total LOS. There was a significant moderately positive correlation between the forward and lateral reach distances (r = 0.606, p = 0.001) and a significant mild correlation between the oblique reach distance and trunk SD (r = 0.44, p = 0.001 and 0.014, respectively). The lateral reach distance had a low correlation with the forward reach distance (r = 0.606, p = 0.001), a moderate correlation with the right lateral reach LOS (r = 0.569, p = 0.001), and a significant mild correlation with the trunk SD (r = 0.396, p = 0.004). The oblique reach distance showed a significant moderate correlation with the forward reach distance, forward reach LOS, and right forward oblique reach LOS (r = 0.44, p = 0.001; r = 0.437, p = 0.002; and r = 0.5, p = 0.001, respectively). There was a significant but mild correlation between the lateral reach distance and total LOS (r = 0.28, p = 0.04 and r = 0.33, p = 0.001, respectively).

**Table 3:** Correlations between reach tests, limits of stability, and anthropometric parameters.

Reach test	Variables	R-value	P value
FRA	LRA in Centimeters	.606**	<0.001
	ORA in Centimeters	.444**	0.001
	Age	−0.138	0.34
	Height in meters	0.265	0.062
	Mass in KG	0.174	0.227
	BMI in KG/Mt Square	0.139	0.337
	Total Limits of Stability Score %	0.102	0.481
	A1 Forward reach LOS %	0.156	0.279
	A2 right forward ODRT LOS %	0.063	0.663
	A3 right Lateral reach LOS %	0.186	0.195
	Total time taken for LOS testing in sec	−0.089	0.538
	Trunk Standard Deviation in degrees	.345*	0.014
LRA	FRA in Centimeters	.606**	<0.001
	ORA in Centimeters	.283*	0.046
	Age	0.07	0.631
	Height in meters	0.123	0.397
	Mass in KG	0.118	0.416
	BMI in KG/Mt Square	0.098	0.496
	Total Limits of Stability Score %	0.15	0.298
	A1 Forward reach LOS %	0.058	0.687
	A2 right forward ODRT LOS %	0.042	0.77
	A3 right Lateral reach LOS %	.569**	<0.001
	Total time taken for LOS testing in sec	0.04	0.784
	Trunk Standard Deviation in degrees	.396**	0.004
ODRA	FRA in Centimeters	.444**	0.001
	LRA in Centimeters	.283*	0.046
	Age	−0.22	0.124
	Height in meters	−0.179	0.214
	Mass in KG	−0.205	0.152
	BMI in KG/Mt Square	−0.136	0.347
	Total Limits of Stability Score %	.330*	0.019
	A1 Forward reach LOS %	.437**	0.002
	A2 right forward ODRT LOS %	.500**	<0.001
	A3 right Lateral reach LOS %	−0.039	0.788
	Total time taken for LOS testing in sec	−0.018	0.899
	Trunk Standard Deviation in degrees	0.03	0.836

**Note:** KG: Kilogram, Mt: Meter, BMI: Body mass index LOS: Limits of stability, FRA: functional reach test average, LRA: Lateral reach average; ORA: Oblique direction reach test average.

**Fig. 7:**
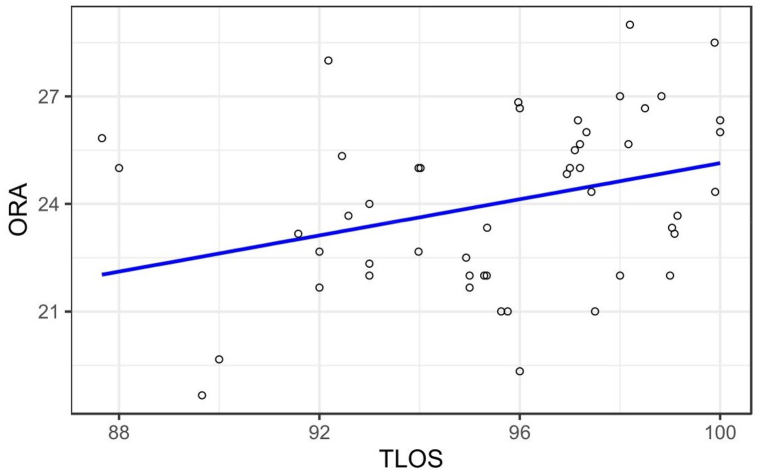
Graph showing the regression analysis relationship between the oblique direction reach test values and total limits of stability.

Table 4, Table 5, Table 6 show the linear regression analysis of the oblique, lateral, and forward reach distances as the dependent variables in relation to the anthropometric parameters, factors of the LOS, and types of the reach test as the independent variables. The regression analysis of the oblique reach distance as a dependent variable demonstrated a significant moderate association with the right forward oblique reach LOS with a beta value of 0.30 and a significance level of 0.02; this finding indicates that the right forward oblique reach LOS influences the oblique reach distance. There was a significant moderate association between the right lateral reach LOS and the lateral reach distance, with a beta value of 0.57 and a significance level of <0.001. The lateral reach distance also demonstrated a moderate association with the forward reach distance, with a beta value of 0.57 and a significance level of 0.001. Moreover, only ODRT showed a moderate relationship with total limits of stability with a beta value of 0.49 at the p-value of 0.04. This relationship is also depicted in Fig. 7.

**Table 4:** Regression analysis keeping oblique direction reach test as a depending factor.

Anthropometric Parameters, factors of limits of stability, and types of reach tests	Unstandardized Coefficients		Standardized Coefficients	t	Sig.
	B	Std. Error	Beta		
Age	−0.50	0.43	−0.14	−1.18	0.25
Height in meters	−15.93	19.88	−0.49	−0.80	0.43
Mass in KG	0.04	0.25	0.29	0.15	0.88
BMI in KG/Mt Square	−0.06	0.68	−0.16	−0.08	0.93
Total Limits of Stability Score %	0.37	0.18	0.49	2.05	0.04*
A1 Forward reach LOS %	0.03	0.03	0.15	1.04	0.31
A2 right forward ODRT LOS %	0.32	0.13	0.30	2.43	0.02*
A3 right Lateral reach LOS %	−0.18	0.07	0.46	−2.63	0.01*
Total time taken for LOS testing in sec	−0.03	0.07	−0.04	−0.35	0.73
Trunk Standard Deviation in degrees	0.23	0.12	0.33	1.85	0.07
FRA in Centimeters	0.17	0.11	0.28	1.62	0.11
LRA in Centimeters	0.13	0.11	0.20	1.16	0.25

**Note**: KG: Kilogram, Mt: Meter, BMI: Body mass index, LOS: Limits of stability, FRA: functional reach test average, LRA: Lateral reach average.

**Table 5:** Regression analysis keeping lateral reach test as a dependent factor.

Anthropometric Parameters, factors of limits of stability, and types of reach tests	Unstandardized Coefficients		Standardized Coefficients	t	Sig.
	B	Std. Error	Beta		
Age	0.90	0.63	0.16	1.43	0.16
Height in meters	−49.94	28.73	−0.96	−1.74	0.09
Mass in KG	0.64	0.36	3.10	1.79	0.08
BMI in KG/Mt Square	−1.71	0.97	−3.04	−1.76	0.09
Total Limits of Stability Score %	−0.31	0.28	−0.26	−1.11	0.27
A1 Forward reach LOS %	0.05	0.04	0.15	1.11	0.27
A2 right forward ODRT LOS %	0.04	0.21	0.03	0.20	0.85
A3 right Lateral reach LOS %	0.36	0.10	0.57	3.75	0.00*
Total time taken for LOS testing in sec	−0.01	0.11	−0.01	−0.09	0.93
Trunk Standard Deviation in degrees	0.30	0.19	0.28	1.62	0.11
FRA in Centimeters	0.47	0.14	0.49	3.31	0.00*
ODRTA in Centimeters	0.28	0.24	0.18	1.16	0.25

**Note:** KG: Kilogram, Mt: Meter, BMI: Body mass index LOS: Limits of stability, FRA: functional reach test average, LRA: Lateral reach average; ORA: Oblique direction reach test average.

**Table 6:** Regression analysis keeping forward reach test as a dependent factor.

Anthropometric Parameters, factors of limits of stability, and types of reach tests	Unstandardized Coefficients		Standardized Coefficients	t	Sig.
	B	Std. Error	Beta		
Age	−0.21	0.65	−0.04	−0.33	0.75
Height in meters	105.86	24.65	1.97	4.30	0.00*
Mass in KG	−1.27	0.32	−5.96	−4.04	0.00*
BMI in KG/Mt Square	3.54	0.84	6.12	4.22	0.00*
Total Limits of Stability Score %	0.47	0.28	0.37	1.68	0.10
A1 Forward reach LOS %	−0.01	0.05	−0.01	−0.10	0.92
A2 right forward ODRT LOS %	−0.35	0.20	−0.20	−1.71	0.10
A3 right Lateral reach LOS %	−0.12	0.11	−0.19	−1.11	0.28
Total time taken for LOS testing in sec	−0.06	0.11	−0.05	−0.52	0.61
Trunk Standard Deviation in degrees	−0.02	0.20	−0.02	−0.10	0.92
ORA in Centimeters	0.39	0.24	0.24	1.62	0.11
LRA in Centimeters	0.48	0.15	0.47	3.31	0.00*

**Note:** KG: Kilogram, Mt: Meter, BMI: Body mass index LOS: Limits of stability, LRA: Lateral reach average; ORA: Oblique direction reach test average.

## 4 Discussion

Reaching is a common function that constantly puts strain on balance. The LOS is required when performing various functional tasks while standing, such as reaching for shelves, grasping a door handle, or operating elevators. These functional tasks occur in different directions, including the forward, lateral, and oblique directions. The ODRT is a newly developed test validated with the FRT and LRT. The validity of this test with COP excursion measurement has yet to be proven. Thus, we sought to establish the concurrent validity of the ODRT with COP excursion measurement, which is considered the gold standard method. We also sought to determine which oblique, forward, and lateral reach distances best correlate with the COP excursion.

The forward reach distance had a non-significant low correlation with the right forward LOS in our study. However, Duncan et al. discovered a moderate correlation between the forward reach distance and COP excursion in their study of 20–87-year-olds [15]. Our study results are inconsistent with those of Duncan et al. which could be attributed to the fact that Duncan et al. studied the correlation over a much wider age span. We found a significant but mild correlation between the forward reach distance and the trunk SD.

In this study, the lateral reach distance demonstrated a moderate but significant correlation with the right lateral reach LOS. Brauer et al. also found a significant correlation between the lateral reach distance and the LOS [17]. The significant correlation between the lateral reach distance and LOS in both studies suggests that the lateral reach distance reflects the LOS in healthy populations. In our study, the lateral reach distance also demonstrated a mild correlation with the trunk SD. This could be attributed to the movement of the lower trunk during lateral flexion, which contributed to the performance of the lateral reach.

The oblique reach distance demonstrated a moderate but significant correlation with the right oblique reach LOS in this study. As, to our knowledge, this is the first study to find a correlation between the oblique reach distance and LOS, we cannot compare our results with those of other studies. Only the oblique reach distance showed a mild correlation with the total LOS herein; neither the forward reach distance nor the lateral reach distance correlated with the total LOS, implying that most functional reaching tasks are performed in the oblique direction rather than in the forward or lateral direction.

We found no correlation between the forward, lateral, and oblique reach distances and the anthropometric parameters. However, Duncan et al. discovered a positive correlation between the forward reach distance and age and height and concluded that age and height influence the forward reach distance [15]. Similarly, in the study by Brauer et al. the lateral reach distance demonstrated a positive correlation with height and age [17]. We included participants in the same age group, mostly of similar height. Duncan et al. included a wider range of age groups, and Brauer et al. also had elderly individuals aged 65–89.

### 4.1 Limitations of the study

Limitations of the current study were that the sample selected was of similar ages. We did not cover all age groups and diseased populations. This study recruited the sample from a single center. Further, the COP excursions for right forward LOS, right lateral LOS, and right oblique reach LOS between men and women were not compared. This study also established the validity in the general population only.

### 4.2 Future recommendations

Forthcoming research should include larger sample sizes. Future studies should include participants from the multicenter. The balance is affected in different age groups due to various neuromuscular disorders and injuries. Therefore, future research should consist of multiple age groups and find the concurrent validity of the ODRT that will be useful in assessing the limits of stability in the clinical setting. We further recommend that future studies concentrate on comparing the LOS in the forward, oblique, and lateral directions between men and women and establish concurrent validity in patient populations.

## 5 Conclusions

All three reach distance measures were symmetrical; however, the forward reach distances were more than oblique and lateral reach distances. Among the measurements, the oblique and lateral reach distances correlated with the COP excursions in the right forward (oblique) and right lateral directions. Furthermore, the oblique reach distance showed a significant moderate correlation with the total LOS, indicating that the ODRT is more functional than the other reach tests in healthy young adults.

Supplementary Materials: All the required materials are available in the manuscript.

## Funding

The authors extend their appreciation to the Deanship of Scientific Research at King Khalid University for funding this work through a large group Research Project under grant number RGP2/328/44.

## Institutional Review Board statement

“The study was conducted in accordance with the Declaration of Helsinki and approved by the Institutional Review Board (or Ethics Committee) of King Khalid University (**ECM#2023 from the ethical research committee of King Khalid University (REC # 2016**–**08**–**29).**

## Ethics statement

“Written informed consent was obtained from all subjects involved in the study,” and “Written informed consent has been obtained from the patient(s) to publish their data, images and information in this paper.”

## Data availability statement

Data is available with the corresponding author mentioned in this research paper.

## CRediT authorship contribution statement

**Jaya Shanker Tedla:** Writing – review & editing, Writing – original draft, Supervision, Software, Project administration, Methodology, Funding acquisition, Formal analysis, Conceptualization. **Devika Rani Sangadala:** Writing – review & editing, Writing – original draft, Software, Resources, Methodology, Investigation, Formal analysis, Data curation. **Ravi Shankar Reddy:** Writing – review & editing, Writing – original draft, Validation, Software, Resources, Methodology, Investigation, Formal analysis, Data curation. **Kumar Gular:** Writing – review & editing, Writing – original draft, Visualization, Software, Resources, Methodology, Investigation, Formal analysis, Data curation. **Venkata Nagaraj Kakaraparthi:** Writing – review & editing, Writing – original draft, Validation, Resources, Methodology, Investigation, Formal analysis, Data curation. **Snehil Dixit:** Writing – review & editing, Writing – original draft, Software, Resources, Methodology, Investigation, Funding acquisition, Formal analysis, Data curation. **Ahlam Mohammed Alamri:** Writing – review & editing, Writing – original draft, Visualization, Validation, Software, Resources, Methodology, Investigation, Formal analysis, Data curation. **Akshatha Nayak:** Writing – review & editing, Writing – original draft, Visualization, Software, Resources, Methodology, Investigation, Formal analysis, Data curation. **Gopal Nambi:** Writing – review & editing, Writing – original draft, Visualization, Software, Resources, Methodology, Investigation, Formal analysis, Data curation. **Bhaskar Reddy:** Writing – review & editing, Writing – original draft, Visualization, Software, Resources, Methodology, Investigation, Formal analysis, Data curation.

## Declaration of competing interest

The authors declare that they have no known competing financial interests or personal relationships that could have appeared to influence the work reported in this paper.

## References

[1] Bergquist R., Weber M., Schwenk M., Ulseth S., Helbostad J.L., Vereijken B. (2019). Performance-based clinical tests of balance and muscle strength used in young seniors: a systematic literature review. BMC Geriatr..

[2] Tedla J.S., Gular K., Reddy R.S., Ferreira A. de S., Rodrigues E.C., Kakaraparthi V.N. (2022). Effectiveness of constraint-induced movement therapy (CIMT) on balance and functional mobility in the stroke population: a systematic review and meta-analysis. Healthcare (Switzerland).

[3] Row J., Chan L., Damiano D., Shenouda C., Collins J., Zampieri C. (2019). Balance assessment in traumatic brain injury: a comparison of the sensory organization and limits of stability tests. J. Neurotrauma.

[4] Schedler S., Brock K., Fleischhauer F., Kiss R., Muehlbauer T. (2020). Effects of balance training on balance performance in youth: are There age differences?. Res. Q. Exerc. Sport.

[5] Lesinski M., Hortobágyi T., Muehlbauer T., Gollhofer A., Granacher U. (2015). Effects of balance training on balance performance in healthy older adults: a systematic review and meta-analysis. Sports Med..

[6] Tedla J.S., SangadalaDR Gular K., Mukherjee D., Gyer G. (2020). Oblique direction reach test: a pilot test to measure limits of stability in oblique direction and its psychometric properties. Chula Med J.

[7] Deshmukh A.A., Ganesan S., Tedla J.S. (2011). Normal values of functional reach and lateral reach tests in indian school children. Pediatr. Phys. Ther..

[8] Mancini M., Horak F.B. (2010). The relevance of clinical balance assessment tools to differentiate balance deficits. Eur. J. Phys. Rehabil. Med..

[9] Umemura G.S., Furtado F., Santos FCd, Gonçalves B.S.B., Forner-Cordero A. (2022). Is balance control affected by sleep deprivation? A systematic review of the impact of sleep on the control of balance. Front. Neurosci..

[10] Dunsky A. (2019). The effect of balance and coordination exercises on quality of life in older adults: a mini-review. Front. Aging Neurosci..

[11] Sibley K.M., Beauchamp M.K., van Ooteghem K., Straus S.E., Jaglal S.B. (2015). Using the systems framework for postural control to analyze the components of balance evaluated in standardized balance measures: a scoping review. Arch. Phys. Med. Rehabil..

[12] Reddy R.S., Tedla J.S., Dixit S., Raizah A., Al-Otaibi M.L., Gular K. (2022). Cervical joint position sense and its correlations with postural stability in subjects with fibromyalgia syndrome. Life.

[13] Alshahrani M.S., Reddy R.S. (2022). Relationship between kinesiophobia and ankle joint position sense and postural control in individuals with chronic ankle instability—a cross-sectional study. Int. J. Environ. Res. Publ. Health.

[14] HoratiuPN Adinam, RemusV (2020). Postural evaluation and physical therapy intervention using Isofree Medical equipment,adapted to dentist- case study. Studia UniversitatisBabes-Boyali, Educatio Artis , Gymnasticase..

[15] Duncan P.W., Weiner D.K., Chandler J., Studenski S. (1990). Functional reach: a new clinical measure of balance. J. Gerontol..

[16] Ferreira S., Raimundo A., Marmeleira J. (2021). Test-retest reliability of the functional reach test and the hand grip strength test in older adults using nursing home services. Ir. J. Med. Sci..

[17] Brauer S., Burns Y., Galley P. (1999). Lateral reach: a clinical measure of medio-lateral postural stability. Physiother. Res. Int..

[18] Newton R.A. (2001). Validity of the Multi-Directional Reach Test: a practical measure for limits of stability in older adults. JGerontol A Biol Sci Med Sci.

[19] Tedla J.S., Asiri F., Alshahrani M.S., Sangadala D.R., Gular K., Rengaramanujam K. (2019). Reference values of functional and lateral reach test among the young saudi population: their psychometric properties and correlation with anthropometric parameters. Med. Sci. Mon. Int. Med. J. Exp. Clin. Res..

[20] Tedla J.S., Sangadala D.R., Gular K., Reddy R.S., Alshahrani M.S., Ahmad I. (2021). Normative reference values for functional, lateral, and oblique direction reach tests in Saudi children aged six to 15 years old and psychometric properties of the oblique direction reach test. Nij J clin Pract.

